# Modification of Ni-Rich FCG NMC and NCA Cathodes by Atomic Layer Deposition: Preventing Surface Phase Transitions for High-Voltage Lithium-Ion Batteries

**DOI:** 10.1038/srep26532

**Published:** 2016-05-26

**Authors:** Debasish Mohanty, Kevin Dahlberg, David M. King, Lamuel A. David, Athena S. Sefat, David L. Wood, Claus Daniel, Subhash Dhar, Vishal Mahajan, Myongjai Lee, Fabio Albano

**Affiliations:** 1 Energy and Transportation Science Division, Oak Ridge National Laboratory, Oak Ridge, TN, USA; 2 Energy Power Systems, LLC, Pontiac, MI, USA; 3 PneumatiCoat Technologies, LLC, Broomfield, CO, USA; 4 Materials Science and Technology Division, Oak Ridge National Laboratory, oak Ridge, TN, USA; 5 Bredesen Center for Interdisciplinary Research and Graduate Education, University of Tennessee, Knoxville, TN, USA; 6 XALT Energy, LLC, Midland, MI, USA; 7 Department of Materials Science and Engineering, University of Michigan Ann Arbor, 2300 Hayward St, Ann Arbor, MI, 48109, USA

## Abstract

The energy density of current lithium-ion batteries (LIBs) based on layered LiMO_2_ cathodes (M = Ni, Mn, Co: NMC; M = Ni, Co, Al: NCA) needs to be improved significantly in order to compete with internal combustion engines and allow for widespread implementation of electric vehicles (EVs). In this report, we show that atomic layer deposition (ALD) of titania (TiO_2_) and alumina (Al_2_O_3_) on Ni-rich FCG NMC and NCA active material particles could substantially improve LIB performance and allow for increased upper cutoff voltage (UCV) during charging, which delivers significantly increased specific energy utilization. Our results show that Al_2_O_3_ coating improved the NMC cycling performance by 40% and the NCA cycling performance by 34% at 1 C/−1 C with respectively 4.35 V and 4.4 V UCV in 2 Ah pouch cells. High resolution TEM/SAED structural characterization revealed that Al_2_O_3_ coatings prevented surface-initiated layered-to-spinel phase transitions in coated materials which were prevalent in uncoated materials. EIS confirmed that Al_2_O_3_-coated materials had significantly lower increase in the charge transfer component of impedance during cycling. The ability to mitigate degradation mechanisms for Ni-rich NMC and NCA illustrated in this report provides insight into a method to enable the performance of high-voltage LIBs.

The USABC (“USABC Goals for Advanced Batteries for EVs - CY 2020 Commercialization”, USCAR, 2015) and U.S. Department of Energy (“Grid Energy Storage”, U.S. Department of Energy, December 2013) have set aggressive battery performance targets for EVs and grid storage applications, respectively^1^,^2^. Such emerging battery applications demand higher voltage and higher energy density than currently commercially available lithium-ion battery (LIB) technologies, with simultaneous improvements to cost, cycle life, and safety^3^,^4^. For a practical system to be compelling at least a 67% reduction in cell cost and more than a 150% increase in volumetric energy density compared to current state-of-the-art Ni-Mn-Co or NMC/graphite and Ni-Co-Al or NCA/graphite cells, while retaining at least 80% of initial capacity after 1000 100% Depth of Discharge (DoD) cycles^1^. As graphite anode materials have a relatively high specific capacity >300 mAh/g and lower cost per unit weight, cathode materials are the limiting component to both energy density and cost per unit energy. Common high-energy commercial layered-structure cathodes like NMC and NCA achieve specific capacities in the range 150–200 mAh/g with moderate charge upper cutoff voltages (UCV) ~4.2 V^5^,^6^; however using higher voltages to realize higher utilization of these materials towards their theoretical capacities ~275 mAh/g causes increased rate of capacity fade and resistance growth (i.e., lower cycle life) symptomatic of cathode-based degradation mechanisms including phase transitions^7^,^8^,^9^,^10^ particle amorphization/pulverization^11^, transition metal dissolution^12^, and electrolyte decomposition^13^.

Electrolyte decomposition at the cathode surface at high voltage, leading to surface passivation and charge transfer impedance growth with negative impact on cycle life, is highly emphasized in the literature; however, the significant contribution of phase transitions to impedance growth has not been as widely recognized, and indeed may be more dominant at higher voltage. Under higher states of delithiation (i.e., high voltage charging), Ni and/or Mn cations are known to migrate to lithium layer tetrahedral and eventually octahedral sites^14^, forming defect spinel and rock salt structures^7^,^8^,^15^. Also at higher voltages, higher metal redox potentials overlap with oxygen 2p energies leading to oxygen anion oxidation and molecular oxygen release^8^,^16^. The resulting oxygen vacancies may also accelerate phase transitions by providing low-energy diffusion pathways for transition metal (TM) migration from TM layer to Li-layer, as is also known for high-voltage lithium-manganese-rich (LMR-NMC) chemistries^14^,^17^,^18^,^19^,^20^. Collectively, the phase transitions create grain boundaries and block Li diffusion pathways and intercalation sites, which cause a charge-transfer impedance increase, and capacity fade. In order to achieve reasonable cycle life utilizing the high capacities gained via high voltage, phase instability is a key problem that must be addressed.

Extensive research on layered-structure oxides has revealed that phase transitions initiate at particle surfaces^7^,^20^,^21^,^22^,^23^,^24^,^25^,^26^. Surface amorphization, cracking, and pulverization have also been observed to initiate at the surface, and have been directly associated with the above reported phase transitions^27^. Therefore, the compounded charge transfer impedance increase at particle surfaces limits the utilization of the bulk, and, moreover, over time and/or cycling structural degradation can propagate into the particle bulk. These phenomena suggest that if the particle surface structure and phase can be preserved, the associated capacity fade and resistance growth may be averted.

Several surface modification and coating methods have been reported to stabilize the surface of layered oxides, including a gradient structure^22^,^23^,^24^, doping^28^, and surface coatings^29^. Al_2_O_3_ surface coatings applied by aqueous, sol-gel, and atomic layer deposition (ALD) have often been reported to mitigate electrolyte decomposition and associated impedance increase^29^,^30^,^31^, and recently researchers have begun to investigate the effects of Al_2_O_3_ surface coatings on “NMC111” phase stability. Kim *et al*. coated the surface of NMC111 particles with Al_2_O_3_ via atomic layered deposition (ALD), and found that charge transfer impedance growth and capacity fade were significantly slower in *half*-*cells* cycled at 1 C/−1 C in 3.0–4.5 V vs. Li^+^/Li^21^. Using selected area electron diffraction (SAED) to characterize the surface structure of a pristine and a surface-coated particle after 100 cycles, they showed that the pristine particle surface became polycrystalline and exhibited spinel cubic rings, whereas the coated particle surface exhibited single-crystal layered structure. Yano *et al*. modified the surface 2–3 nm of NMC111 with Al via a sol-gel technique, retaining a continuous layered structure with LiAlO_2_^32^. They found similar results, that charge transfer impedance growth and capacity fade were significantly decreased in *half*-*cells* cycled at 1 C/−1 C in 2.5–4.7 V vs. Li^+^/Li, however no post-cycling structural analysis was shown to confirm structure stability. While these studies have shown initially promising results for the capability of alumina coatings to stabilize the surface structure, the direct observation of coating chemistry, surface/bulk structure stability, and coating stability during cycling has not been shown. Furthermore, most studies have limited their investigation to coin *half*-*cells* rather than full cells (i.e., vs. graphite anode) with limited Li and electrolyte supply, making the behavior of bare and surface-modified cathodes difficult to assess for commercial applications. Finally, coating studies on layered oxide materials to date have focused mostly on NMC with equal quantities of Ni and Mn, but the capability of surface coatings to stabilize the structure of higher energy but much less stable Ni-rich NMC and NCA has not been shown.

Here we demonstrate the effects of ALD Al_2_O_3_ and TiO_2_ surface coatings on the structure stability and electrochemical performance of layered Ni-rich NMC811^22^ (referred to as NMC here onwards) and NCA, in 2 Ah pouch cells. The motivation of this work is to obtain fundamental insights into the relationship between electrochemical performance and structural stability of coated NMC811 and NCA. Al_2_O_3_ and TiO_2_ surface coatings on NMC and NCA both uniformly covered particle surfaces, yet exhibited distinct electrochemical and structural rearrangement phenomena. Our findings were that i) Al_2_O_3_ coating enhanced capacity retention of NMC-based cells during low and high rate cycling, whereas TiO_2_ coating caused accelerated capacity fade of NMC-based cells during high rate cycling; ii) Al_2_O_3_ and TiO_2_ coatings each enhanced the capacity retention of NCA-based cells during high rate cycling; iii) Al_2_O_3_ coating remained a distinct phase on NMC and NCA particles after cycling at 4.35 V/4.4 V UCV (for NMC and NCA, respectively), preserving the particle surface from phase transformation, associated with a significantly lower rate of charge transfer impedance growth of both NMC and NCA electrodes during 1 C/−1 C cycling; and iv) changes in thickness and uniformity of TiO_2_ coatings on NMC and NCA particle surfaces were observed after cycling at 4.35 V/4.4 V UCV, while the thickness and uniformity of Al_2_O_3_ coatings remained relatively unchanged under these conditions.

To demonstrate the validity and scalability of this approach for use in the manufacture of commercial lithium-ion cells, 2 Ah pouch cells (95 × 64 mm format) were made with ALD-coated and uncoated NMC and NCA cathode materials and used for electrochemical performance and cycling tests with 4.35 and 4.4 UCV, respectively. XALT Energy^33^ experience has shown that 95 × 64 mm pouch cell performance projects reliably to larger formats such as 255 × 255 mm. Prototype large format 255 mm × 255 mm cells are currently under construction in XALT’s Midland Battery Park using a scaled up 450 kg lot of Al_2_O_3_ ALD-coated NMC811 material produced in PneumatiCoat Technologies’ semi-continuous pilot-scale ALD system, and these cells are projected to have an energy density exceeding 500 Wh/L.

## Methods

### Materials

Full concentration gradient (FCG) NMC811^22^ oxide powder was purchased from Posco^®^, and NCA powder was purchased from Toda America^®^. Al_2_O_3_ and TiO_2_ ALD coating on NMC and NCA powders was performed by PneumatiCoat Technologies^34^. Powders were loaded into a batch fluidized bed reactor, heated and dried under vacuum, then iteratively dosed with i) the trimethylaluminum (for Al_2_O_3_) or titanium tetrachloride (for TiO_2_) precursor, ii) inert N_2_, iii) water vapor, and iv) inert N_2_. NMC and NCA uncoated materials are denoted as “NMC-Uncoated” and “NCA-Uncoated”. Al_2_O_3_ and TiO_2_ coated samples are denoted as “X-Y”, where X is NMC or NCA and Y is Al_2_O_3_ or TiO_2_.

Cathode electrodes with ALD-coated and uncoated NMC and NCA were fabricated at XALT Energy’s R&D facility with 15 mg/cm^2^ loading using NMP-based slurries and 92:4:4 active material:Super P:PVDF formulation. Graphite anode electrodes were fabricated with 11 mg/cm^2^ loading using NMP-based slurries and 93:7 active material:PVDF formulation.

The composition of NMC and NCA were determined to be LiNi_0.77_Mn_0.11_Co_0.12_O_2_ and LiNi_0.79_Co_0.16_Al_0.05_O_2_ by inductive coupled plasma atomic emission spectroscopy (ICP-AES) and particle morphology of oxides were evaluated by scanning electron microscopy and energy dispersive x-ray maps were obtained (see the supplementary information). ICP results also showed that Ti was clearly added by ALD coating to NCA-TiO_2_ and NMC-TiO_2_ samples; and Al was clearly added to NMC-Al_2_O_3_; Al addition to NCA-Al_2_O_3_ could not be confirmed by ICP due to the relatively high Al content in the uncoated material.

### Coin cell and 2 Ah pouch cell fabrication

Half coin cells were assembled using a 12 mm diameter disk of NMC and NCA electrodes as the cathode, lithium metal as the anode, a Celgard 2325 separator (19 mm diameter disk), and 1.2 M LiPF_6_ 3:7 EC/DEC as the electrolyte.

2 Ah pouch cells were fabricated by punching 95 × 64 mm electrodes, welding tabs, stacking alternately 9 cathodes and 10 anodes with Z-folded separator, and pouch sealing. The cells were filled with 1.2 M LiPF_6_ 1:9 EC/DEC as the electrolyte before aging and formation.

### Electrochemistry

Rate capability tests from the fresh half coin cells were conducted with different UCV (4.35 V or 4.4 V vs. Li^+^/Li for NMC and NCA, respectively) and LCV of 3.0 V vs. Li^+^/Li at discharge current densities of 0.1 C, 0.2 C, 0.5 C, 1 C, 2 C, 5 C, and 0.1 C (where 1 C = 180 mA/h for NMC and 195 mA/h for NCA electrodes). At each discharge rate, 5 charge-discharge cycles were collected, and for statistics 3 coin cells replicates were used for each electrode test. The capacity fade or cycle life (CL) tests were performed in 2 Ah pouch cells at 0.3 C/−0.3 C and 1 C/−1 C cycling rates with voltage windows of 3–4.35 V for NMC and 3–4.4 V for NCA cells. 2 pouch cell replicates were used for each cathode material and at each rate. NMC cells were each cycled to 80% capacity retention at each rate, and NCA cells were each cycled 300 times.

After completion of 1 C/−1 C CL tests, the 2 Ah pouch cells were disassembled in argon filled glove box, cathodes were extracted, washed with ethanol, and stored in an argon atmosphere for electrochemical and structural characterization. Rate capability tests were performed in half coin cells in a voltage window of 3–4.8 V vs. Li^+^/Li, and discharge current densities of 0.1 C, 0.2 C, 0.5 C, 1 C, 2 C, 5 C, and 0.1 C.

Impedance spectra (400 kHz to 10 mHz, 5 mV perturbation) of each electrode were recorded for fresh and extracted electrodes in half coin cells with a VSP potentiostat (Bio-Logic^®^), after charging to 4.35 V vs. Li^+^/Li for NMC and 4.4 V vs. Li^+^/Li for NCA. Data points at each frequency were averaged by three measurements, and two half coin cell replicates were tested.

### Characterization

A PANalytical X’Pert Pro system with a molybdenum source (λ = 0.76 Å) and automatic divergence and anti-scatter slits operated at 60 kV and 45 mA was used to collect XRD patterns from the pristine and cycled oxide electrodes. A Hitachi HF3300 TEM at 300 kV was used to collect high-resolution TEM images and selected-area electron diffraction (SAED) patterns. TEM specimens of the pristine materials were prepared by dispersing the NMC and NCA powders in ethanol and adding a few drops of the resulting suspension to a holey TEM copper grid. For 1 C/−1 C cycled materials, the powder was scraped from extracted electrodes, ground, dispersed in ethanol, and sonicated for 10–15 minutes. A few drops of the solution were added to the holey Cu grid for TEM observation. Approximately, 15–20 particles were images for each sample to enhance the statistical significance of the observations. SAED were simulated by WebEMAPS (Note: J.M. Zuo and J.C. Mabon, Web-based Electron Microscopy Application Software: Web-EMAPS, Microsc Microanal 10 (Suppl 2), 2004; URL: http://emaps.mrl.uiuc.edu/) using the unit cell of LiMO_2_ structure (M = Ni, Mn, or Co) with 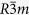 space group, and spinel structure with 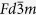 space group as reported elsewhere^35^. Fast Fourier Transformation (FFT) was collected from the high-resolution TEM images. Scanning electron microscopy (SEM) images and energy dispersive X-ray spectroscopy (EDS) data were obtained by Hitachi S4800 FEG-SEM at 20 kV. DC magnetization was measured as a function of temperature, using a Quantum Design Magnetic Property Measurement System. Each sample was first cooled to 5 K in zero field, then a field of 100 Oe was applied and the data were collected from 5 K to 320 K (zero field cooling mode, represented as *ZFC*). The sample was also cooled in the applied field from 320 K down to 5 K, while measuring magnetization (field cooling, represented as *FC*).

## Results

### Electrochemistry

#### Al_2_O_3_ coating improved NMC capacity retention during low rate (0.3 C/−0.3 C) and high rate (1 C/−1 C) cycling

**Figure 1:**
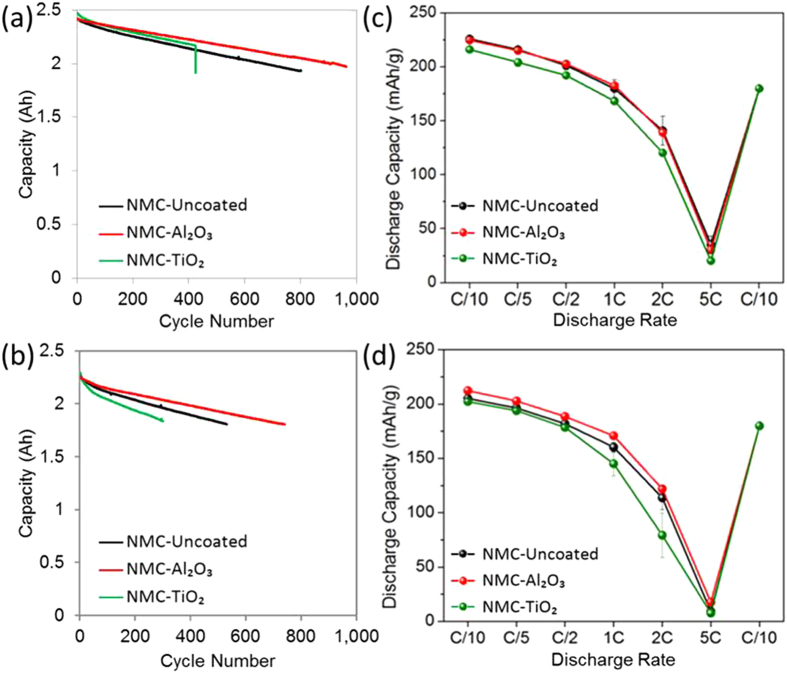
Cycling performance (cycle life) from NMC electrodes in 2 Ah pouch cells at (**a**) low disharge rate (0.3 C/−0.3 C) and (**b**) high discharge rate (1 C/−1 C) with voltage window 3.0–4.35 V. Rate capability of NMC half coin cells with 3.0–4.8 V vs. Li^+^/Li voltage window from (**c**) fresh electrodes and (**d**) electrodes extracted from 2 Ah pouch cells after 1 C/−1 C cycling.

Table 1 summarizes the discharge capacities obtained at beginning of life (BOL) from 2 Ah pouch cells. The BOL 2 Ah pouch cell capacities were similar, 2.41 Ah for NMC-Uncoated, 2.42 Ah for NMC-Al_2_O_3_, and 2.47 Ah for NMC-TiO_2_ electrodes at lower discharge rate (0.3 C). The capacity values decreased with higher discharge rate (1 C) as expected. Figure 1 represents the cycle life performance of 2 Ah pouch cells with coated and uncoated NMC cathodes cycled at low rate (0.3 C/−0.3 C, a) and high rate (1 C/−1 C, b) in a 3–4.35 V voltage window. NMC-Al_2_O_3_ cells had a much lower rate of capacity fade compared to NMC-Uncoated and NMC-TiO_2_ cells at both cycling rates. NMC-TiO_2_ experienced a sudden capacity loss at cycle 425 which terminated the test. At higher rate, the NMC-TiO_2_ had the fastest capacity degradation as compared to uncoated and Al_2_O_3_-coated NMC samples. For all NMC-based cells, coulombic efficiencies were greater than 99.9%, and differences between cells with coated and uncoated cathodes were less than the error limitations of the testing channels used.

#### Al_2_O_3_ and TiO_2_ coatings improved NCA capacity retention during high rate (1 C/−1 C) cycling

**Figure 2:**
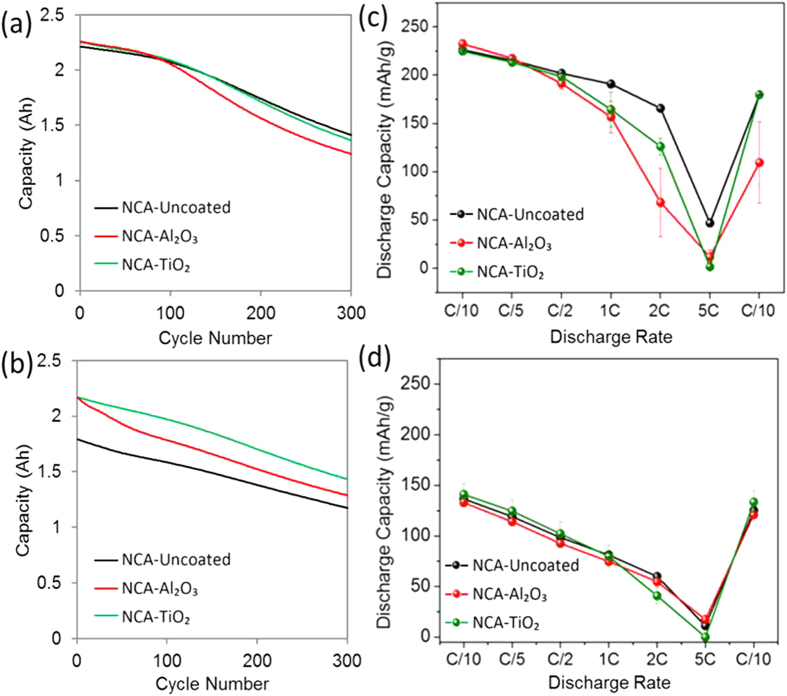
Cycling performance (cycle life) from NCA electrodes in 2 Ah pouch cells at (**a**) low disharge rate (0.3 C/−0.3 C) and (**b**) high discharge rate (1 C/−1 C) with voltage window 3.0–4.4 V. Rate capability of NCA half coin cells with 3.0–4.8 V vs. Li^+^/Li voltage window from (**c**) fresh electrodes and from (**d**) electrodes extracted from 2 Ah pouch cells after 1 C/−1 C cycling.

Table 2 shows the BOL capacities from coated and uncoated NCA cathodes tested in 2 Ah pouch cells. Interestingly, both Al_2_O_3_ and TiO_2_ coatings gave a significantly higher 1 C capacity although the 0.3 C capacities were similar. This may be caused by a higher rate capability of Al_2_O_3_- and TiO_2_-coated NCA, or because certain capacity fade mechanisms occurred immediately in NCA-based cells cycled at 1 C/−1 C. Indeed, the observation of improved rate capability of ALD-coated cathode materials compared to uncoated materials after electrochemical cycling is a common result^21^, and this effect may have been accelerated for NCA. Figure 2 represents the cycle life performance of 2 Ah pouch cells with coated and uncoated NCA cathodes cycled at low rate (0.3 C/−0.3 C, a) and high rate (1 C/−1 C, b) in a 3–4.4 V voltage window. At lower rate, the capacity retention trend was similar up to approximately 70 cycles, with subsequently faster degradation observed for NCA-Al_2_O_3_ up to 300 cycles. At higher rate, the better initial capacity retention with Al_2_O_3_ and TiO_2_ coatings led to 34% and 70% longer cycle life, respectively, relative to the initial capacity of uncoated NCA. During the first 100 cycles, NCA-Al_2_O_3_ had a faster rate of capacity fade than NCA-TiO_2_ and NCA-Uncoated. While coulombic efficiencies of NCA-based cells were very high (>99.8%), and coulombic efficiency differences between cells with coated and uncoated cathodes were generally within the error limitations of the testing channels used, the only statistically significant difference observed was a 0.1–0.2% lower coulombic efficiency for NCA-Al_2_O_3_ compared to NCA-TiO_2_ and NCA-Uncoated during the first 100 cycles.

Rate capability measurements were performed in half coin cells cycled in the voltage window 3–4.8 V vs. Li^+^/Li with fresh electrodes and with electrodes extracted from 2 Ah pouch cells after 1 C/−1 C cycling, and the results are shown in Fig. 1(c,d) for NMC and Fig. 2(c,d) for NCA electrodes. At BOL, NMC-Al_2_O_3_ had a very similar rate capability profile to NMC-Uncoated samples. However, NMC-TiO_2_ showed comparatively lower rate capability in all the NMC materials at similar ALD thicknesses to the Al_2_O_3_ coatings. More significant differences were observed in fresh NCA electrodes, with NCA-Al_2_O_3_ showing worse rate capability than both NCA-TiO_2_ and NCA-Uncoated materials for the thicknesses studied here.

Examining the relative capacity fades evident from half-cell measurements with fresh electrodes and with electrodes extracted from 2 Ah pouch cells cycled at 1 C/−1 C, NMC-Al_2_O_3_ experienced 2.2% 1 C discharge capacity loss in 760 cycles compared to 8.1% in 530 cycles for NMC-Uncoated and 14% in 290 cycles for NMC-TiO_2_. Both NMC-based full cells were cycled to 20% capacity fade; therefore the cathode had a larger contribution to overall capacity fade in NMC-Uncoated cells compared to the NMC-Al_2_O_3_ cells, and cathode-based degradation rates were 5-fold slower with the Al_2_O_3_ ALD coating. Other capacity loss mechanisms should be considered as well, including electrolyte decomposition at the cathode surface, which can result in Li loss and impedance increase; solid-electrolyte interphase (SEI) growth on the anode surface, possibly affected by electrolyte decomposition and transition metal dissolution at the cathode, causing further Li loss and impedance increase. ALD coating on the cathode has been shown to affect both electrolyte decomposition on the cathode and SEI growth on the anode in full cells^31^. However, the rate of non-cathode-based capacity fade with NMC-Uncoated cells (0.022%/cycle) is consistent with NMC-Al_2_O_3_ cells (0.023%/cycle) and NMC-TiO_2_ cells (0.021%/cycle), indicating that these other degradation mechanisms were not significantly affected by the Al_2_O_3_ and TiO_2_ coating thicknesses used in this study.

NCA cathodes were surprisingly seen to experience higher capacity fade than the cells from which they were extracted; NCA-Al_2_O_3_ cathodes experienced 52% 1 C capacity fade in 300 cycles compared to 58% in 300 cycles for NCA-Uncoated, whereas NCA-Al_2_O_3_ cells experienced 40% capacity fade compared to 34% for NCA-Uncoated cells. This may be due to greater instability of the NCA electrodes during the 3–4.8 V vs. Li^+^/Li rate capability cycles prior to the 1 C discharge capacity measurements compared to NMC electrodes. However, NCA-Al_2_O_3_ and NCA-TiO_2_ electrodes both had lower capacity fade than NCA-Uncoated, and similar to the conclusion for NMC, cathode degradation mechanisms likely had a relatively larger contribution to overall capacity fade in NCA-Uncoated cells compared to the NCA-Al_2_O_3_ and NCA-TiO_2_ cells.

#### Al_2_O_3_ coating on NMC and NCA showed lower impedance growth during 1 C/−1 C cycling

**Figure 3:**
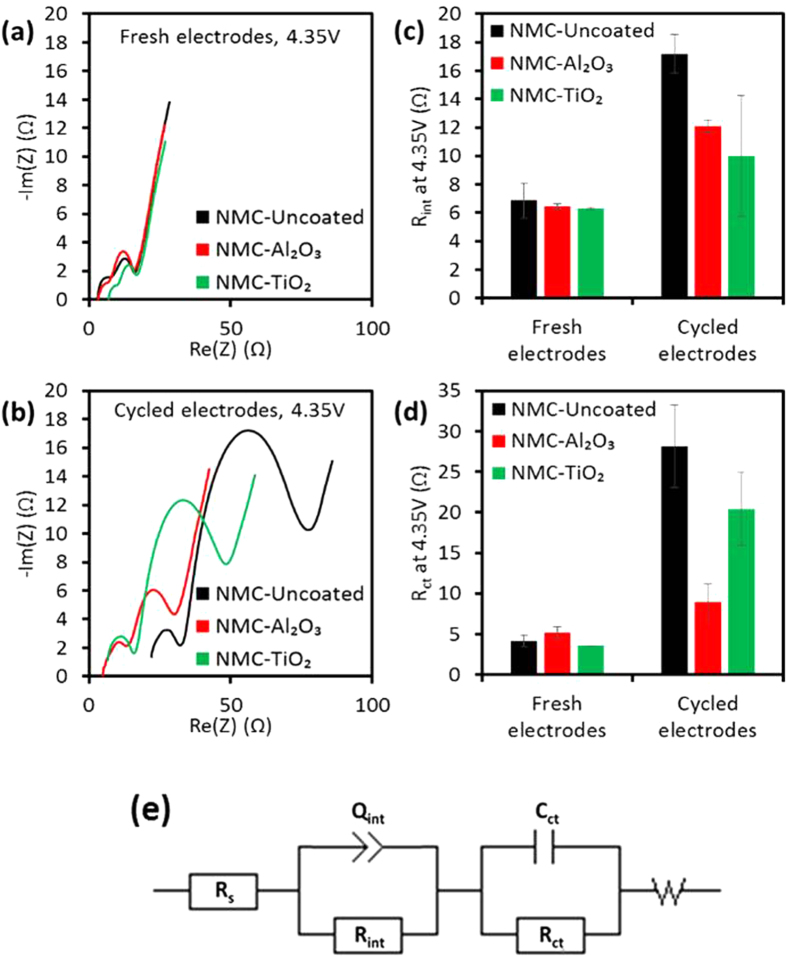
(**a**,**b**) Representative Nyquist plots and (**c**,**d**) impedance components fitted with (**e**) a circuit model for coated and uncoated NMC half-cells after charging to 4.35 V vs. Li^+^/Li for (**a**,**c**) fresh electrodes and (**b**,**d**) electrodes extracted from 2 Ah pouch cells after 1 C/−1 C cycling.

**Figure 4:**
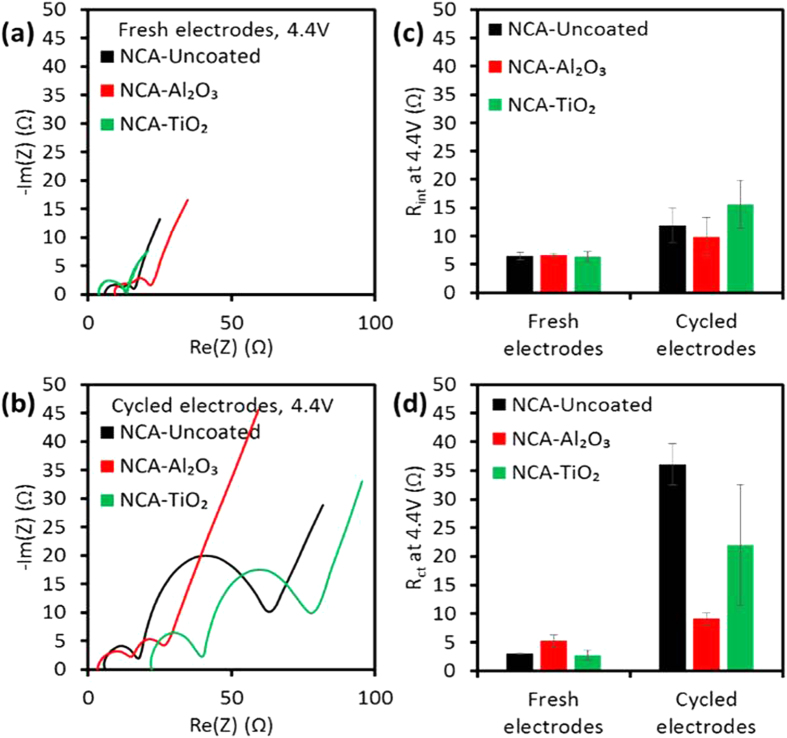
(**a**,**b**) Representative Nyquist plots and (**c**,**d**) impedance components fitted with the circuit model in Fig. 3(e) for coated and uncoated NCA half-cells after charging to 4.4 V vs. Li^+^/Li for (**a**,**c**) fresh electrodes and (**b**,**d**) electrodes extracted from 2 Ah pouch cells after 1 C/−1 C cycling.

Figures 3 and 4 represent the EIS results from fresh NMC and NCA electrodes and from electrodes extracted from 2 Ah pouch cells after 1 C/−1 C cycling, charged in half-cells to 4.35 V and 4.4 V vs. Li^+^/Li, respectively. The EIS data exhibited four resistance components: (1) a time-independent IR resistance, (2) a high-frequency interfacial resistance (R_int_) from the surface passivation layer, (3) an intermediate-frequency charge transfer resistance (R_ct_), and (4) a low-frequency mass transfer resistance (W) in the electrode. R_ct_ is highly dependent on surface structure, and R_ct_ growth at high voltages (>4.3 vs. Li^+^/Li) has been associated with structural instability^7^. These resistance components were calculated using the circuit model shown in Fig. 3. The results revealed that coated cathodes have similar R_int_ and R_ct_ at BOL. All NMC and NCA electrodes experienced an increase in R_ct_ at 4.35 V and 4.4 V vs. Li^+^/Li, respectively, after 1 C/−1 C cycling in 2 Ah pouch cells; however, the Al_2_O_3_ ALD-coated NMC and NCA had much lower growth of R_ct_. Al_2_O_3_ coating gave the slowest rate of R_ct_ increase per cycle, for NCA and particularly for NMC, consistent with the conclusions from half-cell rate capability measurements showing that degradation of NMC and NCA cathodes was slower with the presence of an Al_2_O_3_ ALD coating. These data suggest the principle cathode-based degradation mechanism is surface structure instability causing growth of R_ct_.

The EIS data for NMC-TiO_2_ suggests further complexity. Although NCA-TiO_2_ electrodes extracted from cycled 2 Ah cells had intermediate R_ct_ as well as intermediate 1 C discharge capacities in half-cells compared to NCA-Al_2_O_3_ and NCA-Uncoated, NMC-TiO_2_ had intermediate R_ct_ while also having the fastest rate of capacity loss per cycle compared to NMC-Al_2_O_3_ and NMC-Uncoated. The unique behavior of TiO_2_ ALD coating may be related to the evident modification in its chemistry as shown by TEM analysis presented in a later section, and there may be an additional cathode degradation mechanism with NMC-TiO_2_ that causes Li site loss but relatively less R_ct_ growth. As noted above, the superior capacity retention of NCA-TiO_2_ compared to NCA-Al_2_O_3_ despite the latter’s lowest R_ct_ growth may be related to the lower coulombic efficiency observed with NCA-Al_2_O_3_ cells during the first 100 cycles.

In summary, (i) Al_2_O_3_ ALD coating on NMC particles mitigated the capacity fade during 0.3 C/−0.3 C and 1 C/−1 C cycling; (ii) TiO_2_ ALD coatings on NMC particles accelerated capacity fade, especially during 1 C/−1 C cycling; (iii) Al_2_O_3_ and TiO_2_ ALD coatings on NCA particles increased cell capacities at higher cycling rate (1 C/−1 C); (iv) Al_2_O_3_ and TiO_2_ ALD coatings decreased the rate of R_ct_ growth at 4.35 V and 4.4 V vs. Li^+^/Li for NMC and NCA cathode electrodes, respectively, compared to the uncoated material, with Al_2_O_3_ ALD giving the best performance for the thicknesses selected in this study, and (v) the most important mechanism of cathode capacity loss appeared to be surface structure instability causing charge transfer impedance growth, which was mitigated most by Al_2_O_3_ ALD coating.

### Structural rearrangements

The above electrochemical results provide evidence of the effects of Al_2_O_3_ and TiO_2_ ALD coatings on the electrochemical performance of NMC and NCA cathodes. The cycle life results from 2 Ah pouch cells revealed that the extent of capacity fade at high discharge rates for NMC and NCA-based cells were both improved when cathode particles were coated with Al_2_O_3_ ALD layers. The results for TiO_2_ ALD coatings were different for NMC and NCA, which suggests this coating material can actively interact with these Ni-rich substrates, with NMC experiencing faster capacity fade during high rate cycling with TiO_2_ coating but NCA experiencing significantly slower rate of capacity fade during high rate cycling with TiO_2_ coating. Comparison of cathode rate capabilities and EIS behavior between fresh electrodes and electrodes extracted from 2 Ah pouch cells after 1 C/−1 C cycling strongly suggests that Al_2_O_3_ ALD coating greatly improves the surface structure stability of both NMC and NCA. This section presents a detailed structural investigation to understand this mechanism. Before discussing the structural changes in the cycled cathodes, structure characterization via magnetic susceptibility of fresh, uncoated NMC and NCA powders is shown.

#### NMC and NCA exhibited layered structure, and magnetic ordering observed in pristine NMC suggested unique surface structure

**Figure 5:**
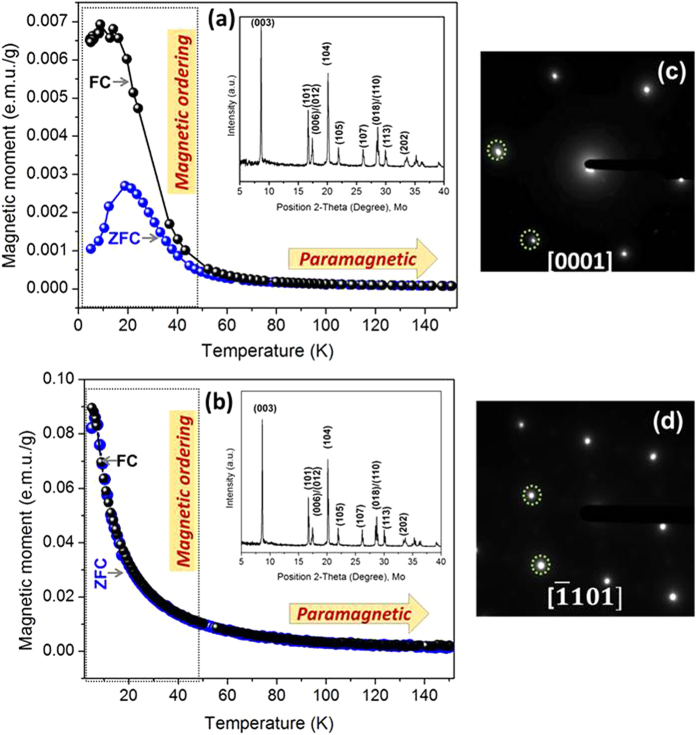
Temperature-dependent magnetic moment at zero filled (filled grey spheres) and field cooling method (filled black spheres) from (**a**) uncoated pristine NMC and (**b**) uncoated pristine NCA at 100 Oe; and SAED from uncoated (**c**) NMC and (**d**) uncoated NCA. The insets in (**a**,**b**) show the corresponding XRD patterns.

The temperature dependent magnetic susceptibility at field cooling (FC) and zero field cooling (ZFC), from NMC and NCA samples (at 100 Oe) along with the XRD patterns are shown in Fig. 5(a,b). All the peaks in the XRD patterns (insets in Fig. a,b) for both NMC and NCA could be assigned to rhombohedral or trigonal (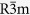 space group, represented as O3 phase). No “cationic ordering” peaks were observed in the XRD patterns of NMC or NCA, as expected. The SAED patterns from the uncoated NMC and NCA materials are shown in the Fig. 5(c,d). The SAED patterns consisted of only fundamental O3-type reflections along the [0001], 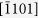 zone axes confirming the O3-type layered structures. At higher temperature (>80 K), magnetic susceptibility from NMC and NCA materials showed the paramagnetic behavior where the magnetic moments of metal ions are randomly oriented again conforming the layered O3-type structure.

At lower temperature (<40 K), in the case of NCA, the FC and ZFC followed the same path down to *T* = 5 K, however in NMC, the FC and ZFC showed a bifurcation at *T* ≈ 50 K. The observation from NCA is very similar to the reported data for layered stoichiometric LiMO_2_ type structure. The bifurcation of FC and ZFC in NMC observed in this study was different than previously reported data and indicate a “magnetic ordering” in the structure. Typically this type of bifurcation is observed in Mn-rich NMC phases such as LiMnO_2_ where onset of “spin-canting”^36^ or lithium-manganese rich NMC materials, where a second Li_2_MnO_3_ phase is present (including a layered LiMO_2_ (O3) phase) and an anti-ferromagnetic interaction between Mn^4+^-O^2−^-Mn^4+^ in Li_2_MnO_3_ phase generates antiferromagnetic ordering^37^,^38^. An antiferromagnetic ordering is not observed if the magnetic moments of M atoms are randomly oriented (in case of O3-type layered structure). The bifurcation in FC and ZFC in NMC in the present study suggests that the atomic arrangements in the layered NMC concentration gradient structure is different than “NMC” with no concentration gradient (see section C for detailed explanation).

#### Al_2_O_3_ ALD coating preserved the particle morphology by protecting the surface during the lithium intercalation/de-intercalation process

**Figure 6:**
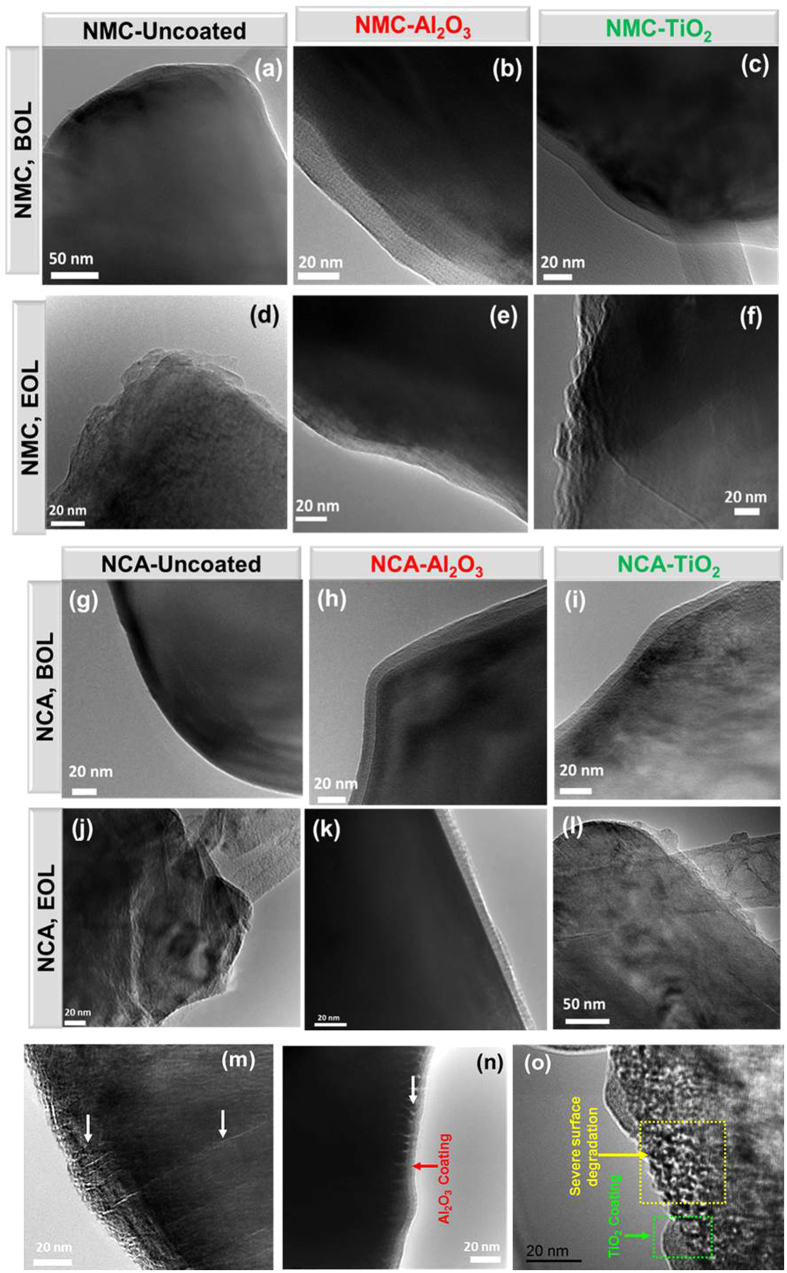
BFTEM images of NMC-Uncoated, NMC-Al_2_O_3_, and NMC-TiO_2_ particles in ((**a**–**c**) respectively) fresh powders and ((**d**–**f**) respectively) extracted from 2 Ah pouch cells after 530, 760, and 290 1 C/−1 C cycles, respectively. BFTEM images of NCA-Uncoated, NCA-Al_2_O_3_, and NCA-TiO_2_ in ((**g**–**i**) respectively) fresh powders and ((**j**–**l**) respectively) extracted from 2 Ah pouch cells after 300 1 C/−1 C cycles. BFTEM images of (**m**) NCA-Uncoated, (**n**) NCA-Al_2_O_3_, and (**o**) NCA-TiO_2_ extracted from full coin cells after 100 1 C/−1 C cycles in a 3–4.8 V vs. Li^+^/Li voltage window.

Figure 6 shows representative bright field (BF) TEM images of pristine NMC (a–c) and NCA (g–i) particles. The NMC-Uncoated and NCA-Uncoated particles showed smooth particle surfaces. Coated NMC and NCA particles showed a distinct, smooth, homogeneous phase on particle surfaces with no interfacial contact gaps, confirming that uniform ALD coatings of Al_2_O_3_ and TiO_2_ were effectively applied on NMC and NCA particles. The thickest applied Al_2_O_3_ and TiO_2_ coating layer thicknesses appeared to be on the order of 10 nm. Figure 6 also shows representative images of coated and uncoated NMC (d–f) and NCA (j–l) particles extracted from cycled 2 Ah pouch cells after 1 C/−1 C cycling. NMC-Uncoated and NCA-Uncoated particles showed corroded surfaces, and cracks had developed in most of the examined particles. In the case of NMC-Al_2_O_3_ and NCA-Al_2_O_3_ particles, the particle surface was preserved in most of the particles, and no cracking and/or particle disintegration was observed. Interestingly, in NMC-TiO_2_ samples, a distinct uniform TiO_2_ coating layer did not remain in many areas. Furthermore, in the NCA-TiO_2_ samples, cracks had developed at some particle surfaces where the remaining presence of a TiO_2_ layer was not apparent. The results showed that the Al_2_O_3_ ALD coating stabilized the particle surface from pulverization and cracking, while TiO_2_ coatings did not form an inert, robust layer phase on these NMC and NCA particles. In order to confirm this hypothesis, full coin cells from NCA-Uncoated, NCA-Al_2_O_3_, and NCA-TiO_2_ were cycled at 1 C/−1 C for 100 cycles at higher voltage (3–4.8 V vs. Li^+^/Li). The representative TEM images from these particles are presented in Fig. 6(m–o). The results revealed that the NCA-Uncoated particle experienced cracking during 1 C cycling (Fig. 6(m)), however the Al_2_O_3_ coating arrests the propagation of cracking (Fig. 6(n)). TiO_2_ coating was observed to have a similar effect where it remained intact (Fig. 6(o)).

#### Al_2_O_3_ ALD coating prevents structural rearrangements at NMC and NCA particle surfaces during cycling, but TiO_2_ ALD coating does not

**Figure 7:**
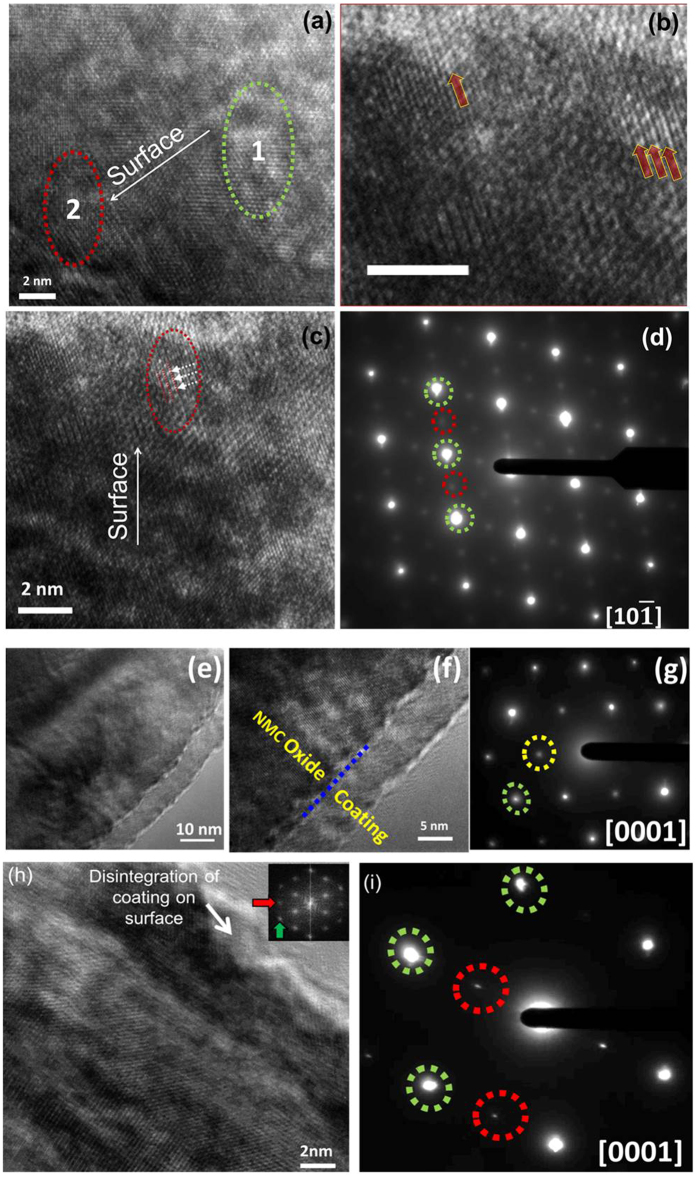
High-resolution TEM images of (**a**–**c**) NMC-Uncoated, (**e**,**f**) NMC-Al_2_O_3_, and (**h**) NMC-TiO_2_ particles extracted from 2 Ah pouch cells after 530, 760, and 290 1 C/−1 C cycles, respectively; and SAED patterns from (**d**) NMC-Uncoated, (**g**) NMC-Al_2_O_3_, and (**i**) NMC-TiO_2_ particles. Scale bar in (**b**) 2 nm.

Figure 7 shows representative high-resolution (HR) TEM images from coated and uncoated NMC samples extracted from 2 Ah pouch cells after cycling at 1 C/−1 C in a 3–4.35 V voltage window. Figure 7(a–c) shows HRTEM images collected for NMC-Uncoated sample. NMC-Uncoated particles exhibited two different phases in single particles marked as Region #1 (towards bulk) and Region #2 (towards surface). As seen in Fig. 7(a), Region #1 was a typical O3-type lattice confirming the layered phase (LiMO_2_-type). However, Region #2 was a spinel-type lattice (AB_2_O_4_-type). The high-magnification view from Region #2 (see Fig. 7(b)) clearly shows the typical spinel-type lattice fringes (indicated by arrows). The spinel-type lattice was observed in several particles, and a second example is shown in Fig. 7(c), where the presence of transition metal atoms in Li-layers was clearly observed. An SAED pattern collected from an NMC-Uncoated particle is shown in Fig. 7(d). The diffraction pattern showed two sets of reflections: bright fundamental O3-type spots correspond to layered trigonal LiMO_2_ phase (indicated by green dotted circles), and faint reflections correspond to a spinel-type phase (indicated by red dotted circles).

HRTEM images of NMC-Al_2_O_3_ particles are shown in Fig. 7(e,f), and the SAED pattern from a representative NMC-Al_2_O_3_ particle is shown in Fig. 7(g). The HR-TEM shows that both the Al_2_O_3_ coating layer and the layered structure of NMC was preserved. The SAED pattern from the [0001] zone axis showed the fundamental O3 reflections from the layered trigonal LiMO_2_ phase (indicated by green dotted circle) and superlattice reflections, often called “forbidden reflections”, that appeared in the middle of the triangle containing O3 spots (indicated by yellow dotted circle). The presence of “forbidden reflections” indicates the presence of Ni in the Li-Layer as reported elsewhere^35^,^39^.

Figure 7(h,i) shows a representative HRTEM image of an NMC-TiO_2_ particle and a representative SAED pattern. The HRTEM image showed the loss of a distinct TiO_2_ coating phase from the particle surface; the FFT processed from the corresponding HRTEM image showed the fundamental reflections (indicated by green arrow), and the spinel reflections (indicated by red arrow). The SAED pattern was in agreement with these results, where fundamental layered O3 spots from the trigonal phase (indicated by green dotted circles) and faint spinel spots (indicated by red dotted circles) were observed. Spinel-type reflections were observed in most of the examined NMC-TiO_2_ particles.

**Figure 8:**
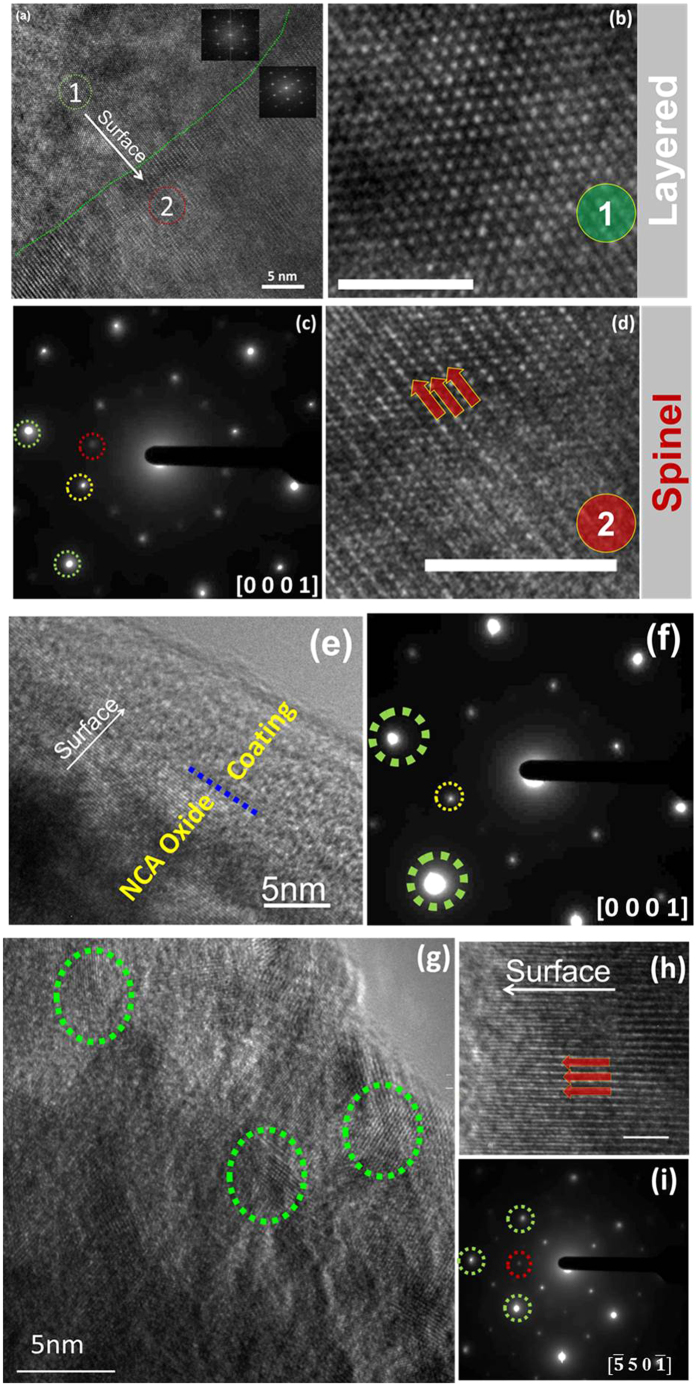
Representative HRTEM images of (**a**,**b**,**d**) NCA-Uncoated, (**e**) NCA-Al_2_O_3_, and (**g**,**h**) NCA-TiO_2_ particles extracted from 2 Ah cells after 300 1 C/−1 C cycles; and SAED patterns from representative (**c**) NMC-Uncoated, (**f**) NMC-Al_2_O_3_, and (**i**) NMC-TiO_2_ particles.

Figure 8(a,b) shows a representative HRTEM image and SAED pattern for NCA-Uncoated particles extracted from 2 Ah pouch cells after 300 1 C/−1 C cycles. Lattice fringes from an O3 layered phase (Region #1) and spinel phase (Region #2) were separated by a distinct boundary. FFT from a magnified view from Region #1 (Fig. 8(c)) confirmed the O3 lattice (hexagonal pattern), whereas FFT from a magnified view from Region #2 (Fig. 8(d)) clearly showed the spinel lattice. The SAED pattern for this region (Fig. 8(c)) showed fundamental O3 reflections from the layered LiMO_2_ phase (indicated by green dotted circles), spinel-type reflections (indicated by red dotted circle), and “forbidden reflections” (indicated by yellow dotted circle).

Figure 8(e) shows a representative HRTEM image of a NCA-Al_2_O_3_ particle extracted from a cycled 2 Ah pouch cell. Similar to NMC-Al_2_O_3_, NCA-Al_2_O_3_ sample does not show any phase transition, and the layered lattice was observed in most of the analyzed particles. The Al_2_O_3_ coating layer was preserved, however in these images, the thickness of the Al_2_O_3_ coating appeared to have decreased during cycling as compared to the cycled NMC-Al_2_O_3_ particles. This observation merits a further ALD film thickness dependence study, which is currently underway. A representative SAED pattern for an NCA-Al_2_O_3_ particle (Fig. 8(f)) showed the fundamental O3 spots from the layered LiMO_2_ phase (indicated by green dotted circles) and “forbidden reflections” (indicated by yellow dotted circle).

In contrast, NCA-TiO_2_ showed very different behavior compared to NCA-Al_2_O_3_ and NCA-Uncoated. In some of the NCA-TiO_2_ particles, the coating remained intact over the entire particle surface, however in other particles the coating had disappeared. On particles where the coating had disappeared, lattice fringes with different orientations were observed near the surface as compared to the bulk (indicated by green dotted circles in Fig. 8(g)). A second representative HRTEM image of a NCA-TiO_2_ particle showed the absence of a distinct TiO_2_ coating layer, and a spinel-type phase on the surface (Fig. 8(h)). A representative SAED pattern (Fig. 8(i)) from NCA-TiO_2_ showed fundamental O3 spots from the layered trigonal LiMO_2_ phase (indicated by dotted green circles) and spinel spots (indicated by dotted red circle).

## Discussion

The results above show that ALD surface coating, particularly Al_2_O_3_ coating, stabilized the NMC and NCA phase structures at particle surfaces, causing slower charge transfer impedance growth in cathode electrodes and higher capacity retention of cells with both NMC and NCA cathodes during high rate cycling. The fresh NMC and NCA cathode materials have a layered structure in which lithium atoms occupy octahedral sites (Li_Oct_) in lithium layers and TM atoms (Co, Mn, Ni, Al) occupy octahedral sites (TM_Oct_) in transition metal layers in a hexagonal structure (O3-type) with trigonal symmetry (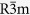 space group). The structure of NMC is slightly different than NCA due to the concentration gradient distribution of Mn and Ni between surface towards the bulk in NMC. Specifically, the observation of magnetic ordering (antiferromagnetic behavior) in the magnetic susceptibility data indicates the presence of Mn-rich clusters (amorphous/crystalline) of Li_2_MnO_3_ or LiMnO_2_ likely in surface regimes where Mn concentration was higher. There was no sign of “cationic ordering” peaks or “C2/m” reflections in XRD and SAED patterns, respectively. Therefore the observation of antiferromagnetic behavior in the magnetization data suggests that the clusters were amorphous, and therefore were not able to be detected in XRD and SAED. However, detailed investigation is needed to confirm this hypothesis. The Mn-rich phase likely concentrated at FCG NMC particle surfaces in this study is very little, so that phase restructuring only contributes towards the impedance rise, and may not cause cell-voltage fade as it occurs in Li-Mn-rich oxides (LMR oxides).

During charging and discharging, Li_Oct_ cations are de-intercalated from and intercalated into, respectively, the host NCM/NCA structure. Charging to higher UCV (≥4.3 V in full cells) may induce oxygen loss selectively from the surface that decreases the bonding interaction among the metal atoms and subsequently causes metal cations to migrate from the metal layer to the lithium layer, rearranging the host oxide structure and triggering structural transformation. The results discussed above show the degradation phenomena in particle morphology and phase transformations that occur in NMC/NCA-Uncoated samples. This result is surprising for NMC-Uncoated given that FCG NMC materials used in this work have been engineered to provide a more stable surface structure than conventional NMC materials with a homogeneous metal distribution, based on the relative stability of Mn^4+^ compared to Ni^4+^ and Co^4+^ to oxygen release, electrolyte oxidation, and phase transformations^22^,^24^. But Figs 6(d) and 7(a–d) clearly show that surface degradation occurred in full 2 Ah pouch cells during 1 C/−1 C cycling in the 3–4.35 V voltage window, including layered-to-spinel phase transitions. Sun *et al*. reported that no cubic phase could be detected via SAED in concentration gradient NMC (with similar Ni content to the NMC in this study) after 50 0.2 C/−0.2 C cycles with 4.3 V vs. Li^+^/Li UCV in half cells (equivalent to approximately 4.2 V in a full cell with graphite anode) at 55 °C, while conventional distribution NMC had a prevalent cubic phase at the particle surface after cycling. However, the conditions used in that study^24^ were much less severe than the 4.35 V UCV, 1 C/−1 C rate, and 530 cycles used in the current study for NMC-Uncoated in 2 Ah pouch cells. Moreover, in the previous studies^22^,^23^,^24^, the capacity fade rate of gradient NMC increased with UCV in the range 4.3–4.5 V vs. Li^+^/Li in half cells at similar C-rates and temperatures, although 4.3 V vs. Li^+^/Li was the only UCV for which structure analysis was performed after cycling.

ALD surface coatings on NMC and NCA particles may restrict oxygen release at high voltages, thereby stabilizing their surface structure. However, different coatings must be expected to have unique interactions with different cathode chemistries, and coatings may or may not suppress phase transformations. Structural rearrangement and transformation occurred in the cases of NCA-Uncoated, NMC-Uncoated, NCA-TiO_2_, and NMC-TiO_2_ samples, whereas the structures of NCA-Al_2_O_3_ and NMC-Al_2_O_3_ remained mostly layered and had the lowest growth of charge transfer impedance. The unique stabilizing effect of Al_2_O_3_ coating compared to TiO_2_, which are each amorphous in the as-deposited state, is likely associated with its capability to maintain intimate bonding with the oxide surface while remaining a distinct phase over hundreds of cycles, excluding transition metal cations from the surface. It should be noted that the TiO_2_ ALD coating did indeed stabilize the surface structure locally where it remained a distinct phase, particularly for NCA, corresponding to intermediate charge transfer impedance growth, further suggesting that the key difference between Al_2_O_3_ and TiO_2_ coatings is the sustained robustness of a distinct coating phase. A unique interaction between NMC and TiO_2_ was suggested by the result that NMC-TiO_2_ electrodes extracted from 2 Ah pouch cells cycled at 1 C/−1 C had relatively low charge transfer impedance but also relatively low capacity, suggesting a Li site loss mechanism in addition to the surface structural degradation mechanisms that explain other results. It can be hypothesized that a distinct TiO_2_ coating phase may be lost due to a complex process of local lithiation of the coating phase as has been shown for Al_2_O_3_^40^, Ti cation migration into the substrate structure, and/or the formation of new phases that may occur in the TiO_2_ ALD coating itself. Carefully designed *in-situ* electrochemical experiments and high resolution *ex-situ* atomic mapping and diffraction are required to characterize the evolved surface chemistry. The value of understanding any underlying phenomena would be to aid in the design and optimization of more complex or multifunctional ALD coatings that are tailored to achieve the performance metrics of high energy cathode materials across a spectrum of end-use applications.

Cathode structural instability and associated impedance growth is accelerated by higher cycling rates due to the relatively greater state of surface delithiation at particle surfaces at end of charge^12^, as well as the higher mechanical stresses^11^,^27^. Therefore at higher cycling rates, similar to the effect of higher voltages, cathode degradation mechanisms will constitute a larger component of total cell capacity loss. This is consistent with the results for coated NMC cathodes, where Al_2_O_3_ ALD coatings increased cycle life by 25% and 40% during lower rate and higher rate cycling, respectively; and also coated NCA cathodes, where both Al_2_O_3_ and TiO_2_ coating increased cycle life during higher rate cycling but had little or negative effect on cycle life during lower rate cycling. The result that TiO_2_ coating on NCA had a positive effect only for high rate cycling while TiO_2_ coating on NMC had a negative effect even at higher rate suggests that surface phase transitions and particle cracking of NCA was prevented during initial high rate cycles while the TiO_2_ coating phase was more intact, whereas these cathode degradation processes occurred more slowly for NMC. Moreover, TEM analysis showed that the TiO_2_ coating phase was more stable on NCA particles than on NMC particles, and as noted above, a unique interaction between TiO_2_ and NMC may have caused an additional Li site loss degradation mechanism not observed with Al_2_O_3_-coated and uncoated NMC. Finally, particle cracking was clearly more severe for NCA particles than NMC particles, as seen in TEM images of samples extracted from NMC-Uncoated electrodes after 530 cycles (Fig. 6(d)) and NCA-Uncoated electrodes after 300 cycles (Fig. 6(j)), and the capability of TiO_2_ coating to mitigate crack propagation during initial cycles for NCA may be more important than mitigating surface phase transitions.

Overall, the structural and phase stability of Al_2_O_3_ ALD-coated NMC and NCA was evident in the low rate of charge transfer impedance growth during cycling, consistent with the preservation of a single phase and lack of grain and/or phase boundaries at particle surfaces. Interestingly, the fact that Al_2_O_3_ coating stabilized the surface structure of gradient NMC at high UCV (approximately 4.45 V vs. Li^+^/Li) and high rate (1 C/−1 C) for 760 cycles shows that a simple Al_2_O_3_ ALD coating has a much more significant impact on structure stability than the gradient metal distribution within the highly-engineered substrate cathode particles. Capacity measurements for NMC-Uncoated and NMC-Al_2_O_3_ electrodes extracted from 2 Ah pouch cells cycled at 1 C/−1 C (530 and 760 cycles, respectively) indicated that 8.1% of total capacity fade over 530 cycles in NMC-Uncoated cells was due to the cathode, compared to only 2.2% of total capacity fade over 760 cycles in NMC-Al_2_O_3_ cells, a 5-fold improvement.

## Conclusions

**Figure 9:**
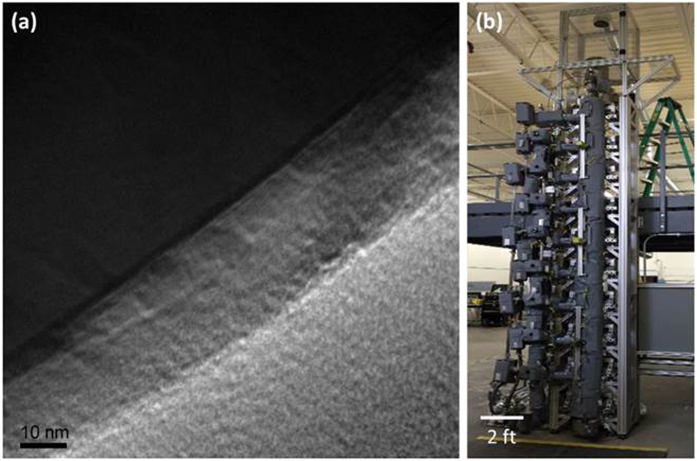
(**a**) Representative BFTEM image of Al_2_O_3_-coated NMC811 particles in a powder specimen from a 450 kg lot produced in (**b**) PneumatiCoat Technologies’ 20 ft high-throughput semi-continuous system, planned for use in XALT large format 255 mm × 255 mm pouch cells.

Surface structure degradation mechanisms including surface-initiated phase transitions and crack propagation cause charge transfer impedance growth during electrochemical cycling, particularly for Ni-rich NMC and NCA at high C-rate and high UCV, and ultimately capacity fade. This work demonstrates the capability of Al_2_O_3_ surface coatings applied by ALD on Ni-rich NMC and NCA particles to significantly slow or prevent these mechanisms. Although NMC811 with a concentration gradient experienced layered-to-spinel phase transitions at particle surfaces when cycled in 2 Ah pouch cells with 4.35 V UCV at 1 C/−1 C, Al_2_O_3_-coated NMC particles exhibited only layered structure even after 40% more cycles to the same capacity loss. Likewise, Al_2_O_3_-coated NCA particles exhibited only the layered phase after 300 1 C/−1 C cycles and gave 34% longer cycle life than uncoated NCA with 4.4 V UCV in 2 Ah pouch cells, whereas uncoated NCA particles experienced extensive layered-to-spinel phase transitions and particle cracking during the same number of cycles. Based on the promising results of this study for NMC-Al_2_O_3_, 450 kg of NMC-Al_2_O_3_ powder was manufactured in PneumatiCoat Technologies’ high-throughput semi-continuous ALD system (Fig. 9(b)), which is currently being scaled to achieve a 1,000+ metric ton per year capacity in a single system. The powder will be used to produce XALT large format 255 mm × 255 mm pouch cells. The representative TEM images of the scaled up ALD-coated material shown in Fig. 9(a) showed the same high quality coating as for the smaller NMC-Al_2_O_3_ batches used to produce 2 Ah pouch cells.

While the benefits of Al_2_O_3_ ALD coating for NMC are remarkable, and the same structural stabilizing effects are present for Al_2_O_3_-coated NCA, the benefits of Al_2_O_3_ coating and particularly TiO_2_ coating on NCA particles for high rate cycling requires further study. For example, neutron diffraction analysis is planned to confirm the nature of new phases that may have formed in NMC-TiO_2_ and NCA-TiO_2_ samples, and to quantify the amount of layered and spinel phases in coated and uncoated materials after cycling. These results will be presented in a future publication.

This work highlights the dominant effects of surface chemistry on active material performance. Continued development of surface coatings promises to open new pathways to tune properties and performance of a wide range of active materials. The new functionality of surface coatings to stabilize surface structure of Ni-rich NMC811 and NCA demonstrated in this study appears to have greater impact than metal composition or distribution, and is critical for the ability to cycle these Ni-rich materials with higher upper cutoff voltages to achieve significantly greater energy densities without sacrifice to cell cost or cycle life.

### Phase transformation mitigation strategies for layered oxides by ALD coatings (Broader Impact)

The atomic layer deposition (ALD) technique has served as the most versatile coating technology for oxides. In the realm of energy storage research, it is important to maintain the coating homogeneity on the electrode oxide particle in order to restrict the undesired phenomena such as phase transformations, oxygen gas release, particle cracking, and transition metal ion dissolution that occur at the oxide particle surface that eventually diffuse towards the bulk during subsequent charge, discharge cycles. The ALD coating technique offers the unique strategy to coat the oxide particle homogenously and uniformly to mitigate these degradation phenomena. This study shows how the ALD coatings were indispensable in restricting phase transformation in layered LiMO_2_ (M = Mn/Al, Co, Ni). This strategy can be extended to other high-voltage, and high capacity electrodes for high-energy-density lithium-ion battery applications such as Li-Mn-rich NMC and polyionic oxides. However, selection of the coating chemistry, and understanding the interfacial effect of coating and the active oxide particles are vital as described in this present work. Therefore, this research demands a simultaneous investigation by computation and experiments to obtain the deeper insights into the effect of ALD coatings on lithium-ion battery performance.

## Additional Information

**How to cite this article**: Mohanty, D. *et al*. Modification of Ni-Rich FCG NMC and NCA Cathodes by Atomic Layer Deposition: Preventing Surface Phase Transitions for High-Voltage Lithium-Ion Batteries. *Sci. Rep.* **6**, 26532; doi: 10.1038/srep26532 (2016).

## Supplementary Material

Supplementary Information

## Figures and Tables

**Table 1:** The beginning of life (BOL) discharge capacity from NMC 2 Ah cells at different C-rates and in different voltage windows.

Cell	2 Ah pouch cell capacity at beginning of life (Ah ± std. dev.)	
C-rate	C/3/−C/3	1 C/−1 C
NMC-Uncoated	2.41 ± 0.01	2.27 ± 0.02
NMC-Al_2_O_3_	2.417 ± 0.003	2.262 ± 0.007
NMC-TiO_2_	2.50 ± 0.03	2.27 ± 0.04

**Table 2:** The BOL discharge capacities of NCA electrodes at different C-rates and in different voltage windows.

Cell	2 Ah pouch cell capacity at beginning of life (Ah ± std. dev.)	
C-rate	0.3 C/−0.3 C	1 C/−1 C
NCA-Uncoated	2.18 ± 0.04	1.83 ± 0.06
NCA-Al_2_O_3_	2.24 ± 0.02	2.13 ± 0.04
NCA-TiO_2_	2.17 ± 0.12	2.10 ± 0.11
