# Supplementary material for: Modification of Ni-Rich FCG NMC and NCA Cathodes by Atomic Layer Deposition: Preventing Surface Phase Transitions for High-Voltage Lithium-Ion Batteries
Source: Sci Rep. 2016 May 26;6:26532. doi: 10.1038/srep26532 (PMC4880920; doi:10.1038/srep26532)
Supplement: Supplementary Information [file srep26532-s1.pdf]

Electronic Supplementary Information

Modification of Ni-Rich FCG NMC and NCA Cathodes by Atomic Layer Deposition: Preventing Surface Phase Transitions for High-Voltage Lithium-Ion Batteries

Debasish Mohanty, [1,*] *Kevin Dahlberg,* [2] *David M. King,* [3] *Lamuel A. David,* [1] *Athena S. Sefat,* [4] *David L. Wood III,* [1,5] *Claus Daniel,* [1,5] *Subhash Dhar,* [2,6] *Vishal Mahajan,* [6] *Myongjai Lee,* [6] *Fabio Albano*[2,6 *]

[1]*Energy and Transportation Science Division, Oak Ridge National Laboratory, Oak Ridge, TN, USA*
[2]*Energy Power Systems, LLC, Pontiac, MI, USA*
[3]*PneumatiCoat Technologies, LLC, Broomfield, CO, USA*
[4]*Materials Science and Technology Division, Oak Ridge National Laboratory, oak Ridge, TN, USA*
[5]*Bredesen Center for Interdisciplinary Research and Graduate Education, University of Tennessee, Knoxville, TN, USA*

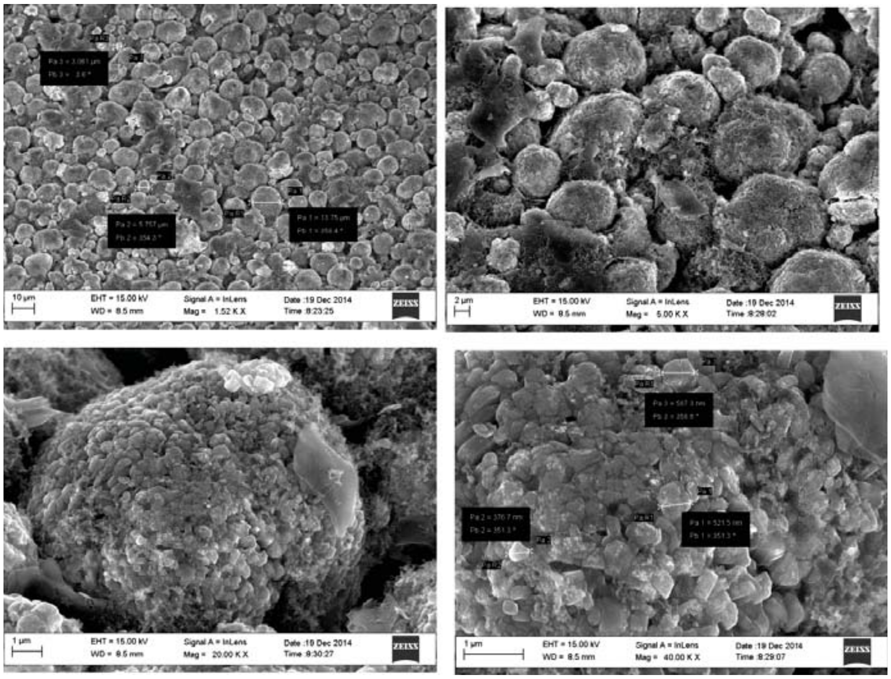

Figure 1: SEM images from NMC-Uncoated sample

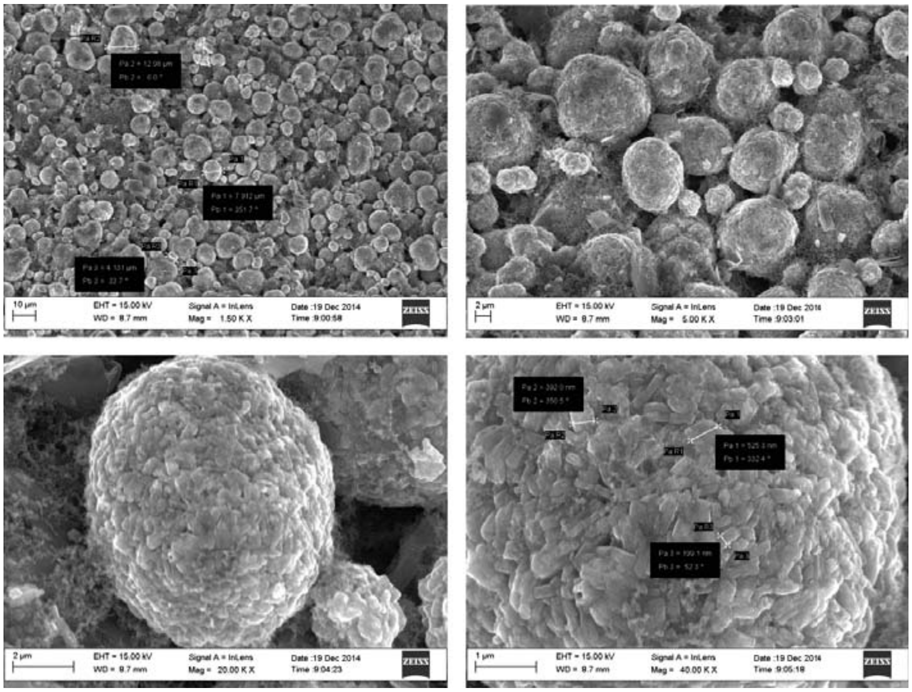

Figure 2: SEM images from NMC-Al2O3 sample

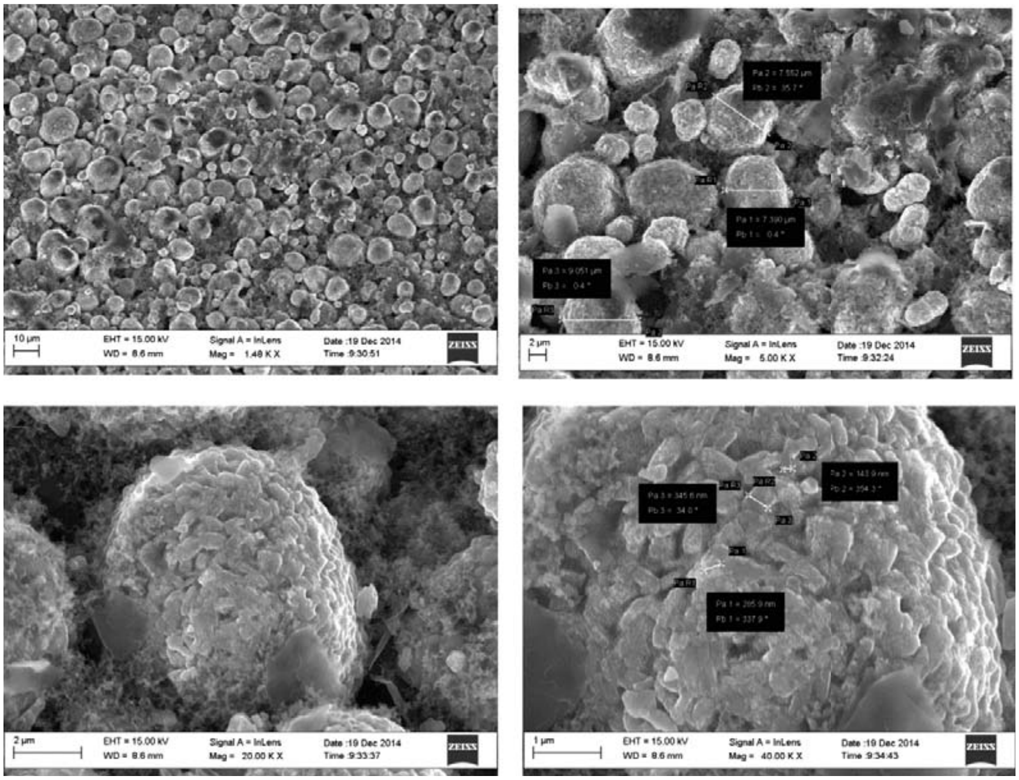

Figure 3: SEM images from NMC-TiO2 sample

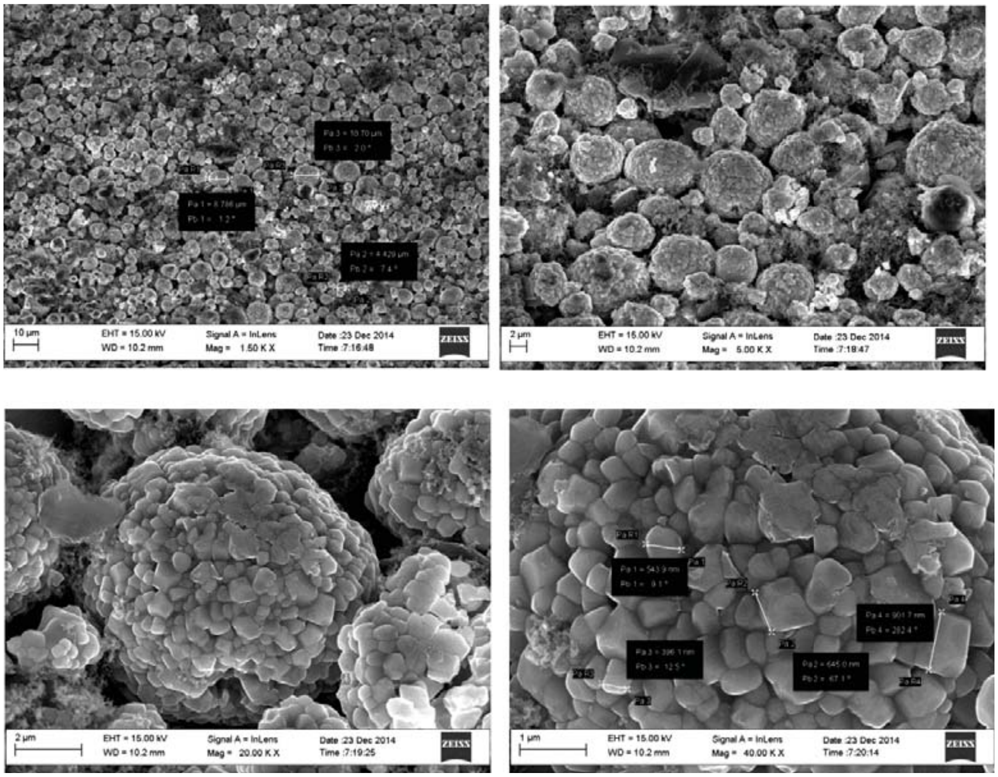

Figure 4: SEM images form NCA-Uncoated sample

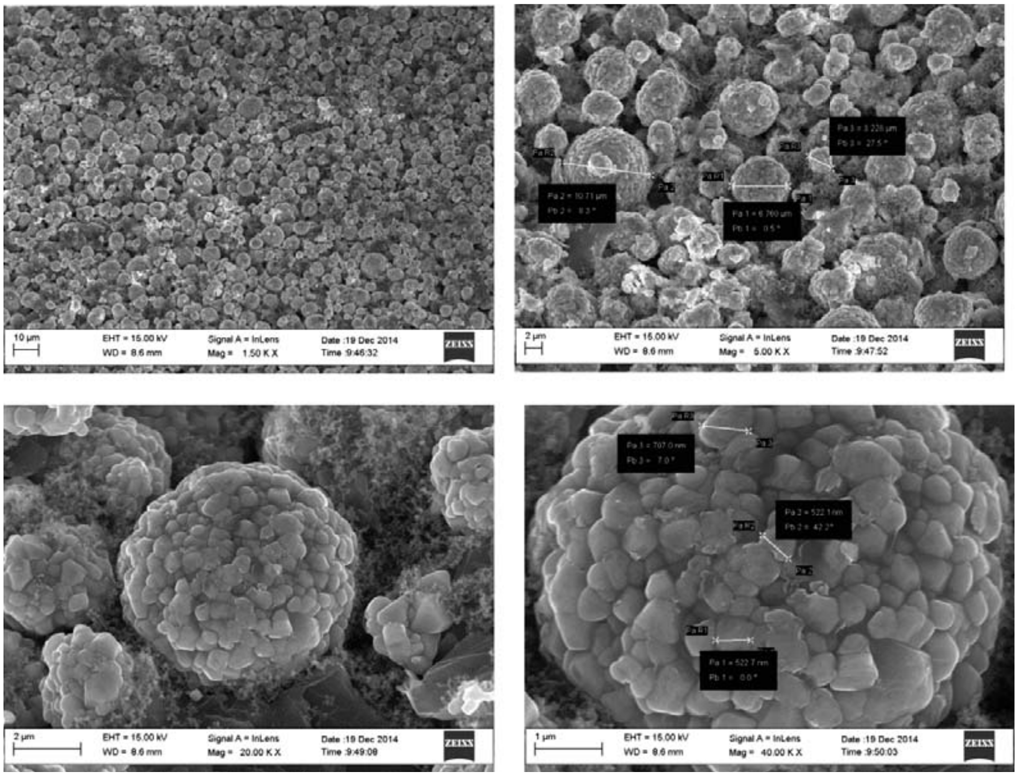

Figure 5: SEM images from NCA-Al2O3 sample

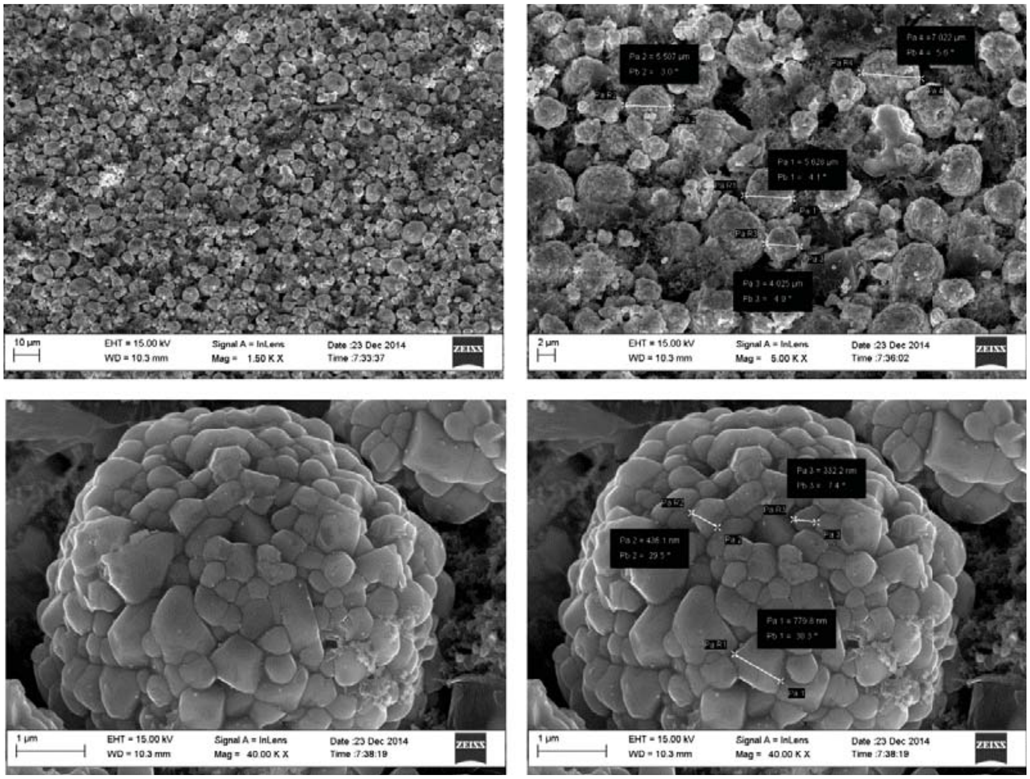

Figure 6: SEM images from NCA-TiO2 sample

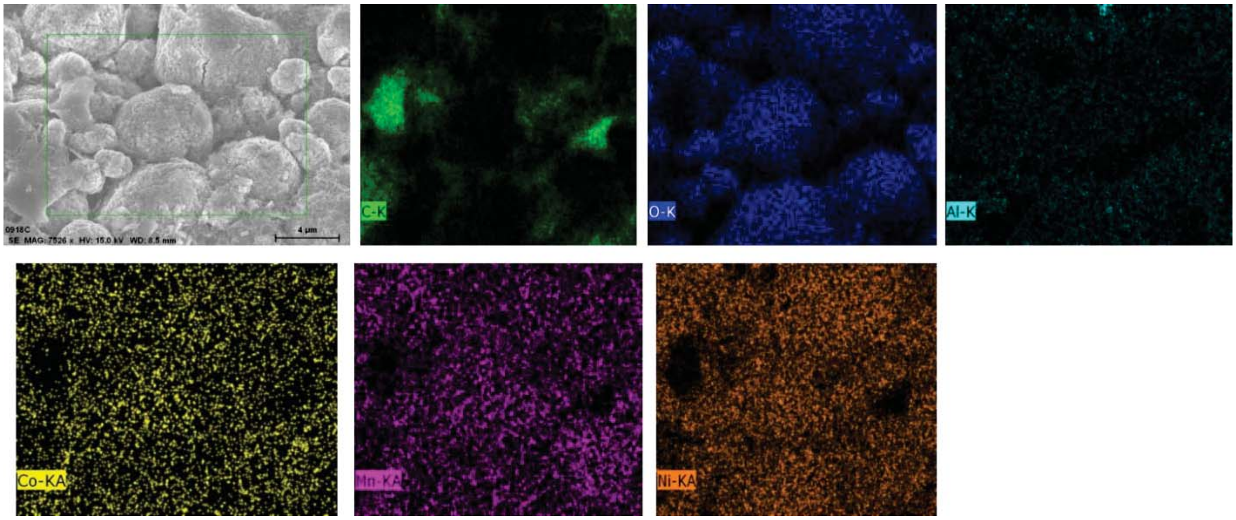

Figure 7: x-ray mapping from NMC-Uncoated sample

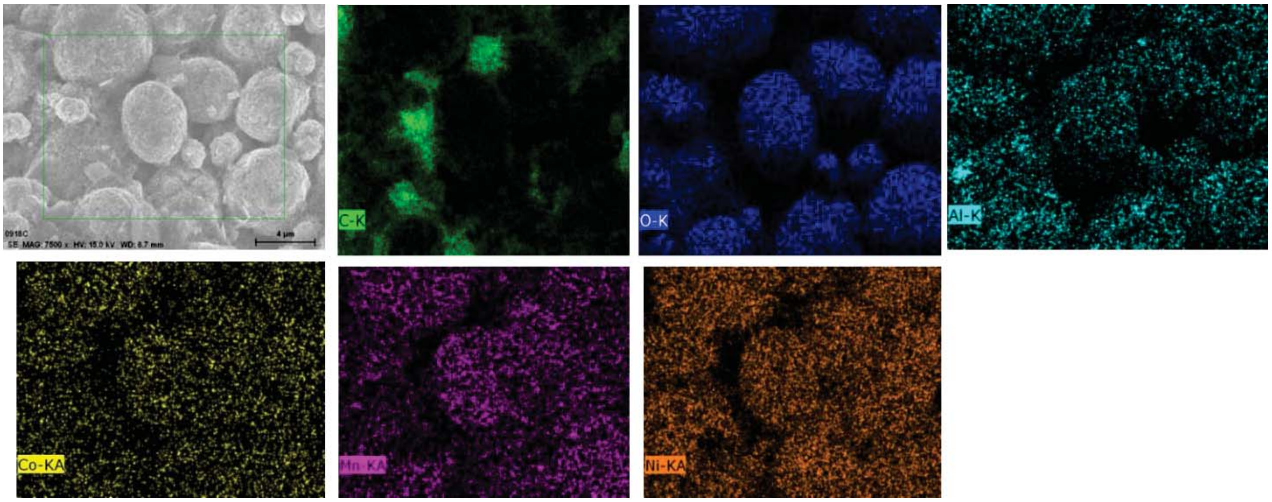

Figure 8: x-ray mapping from NMC-Al2O3 particle

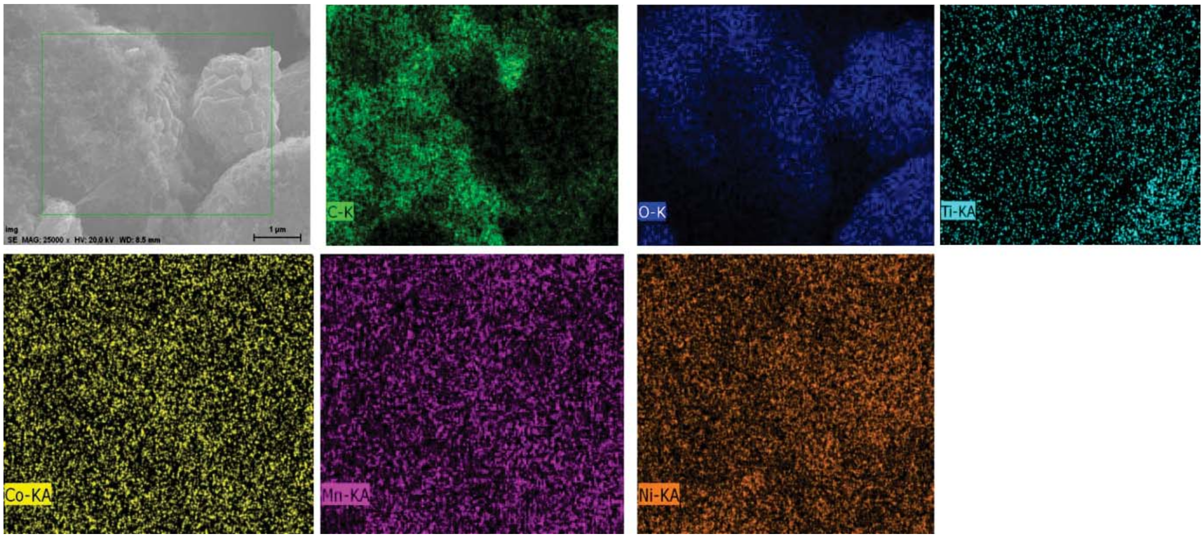

Figure 9: x-ray mapping from NMC-TiO2 particle

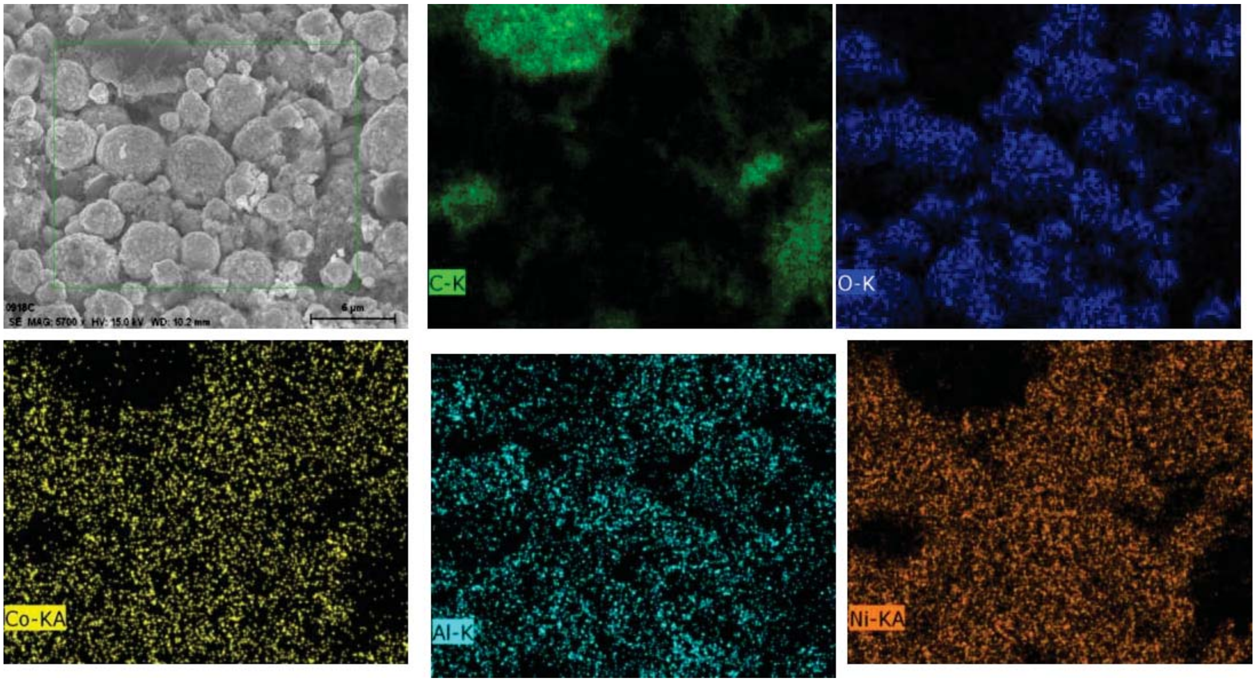

Figure 10: x-ray mapping from NCA-uncoated sample

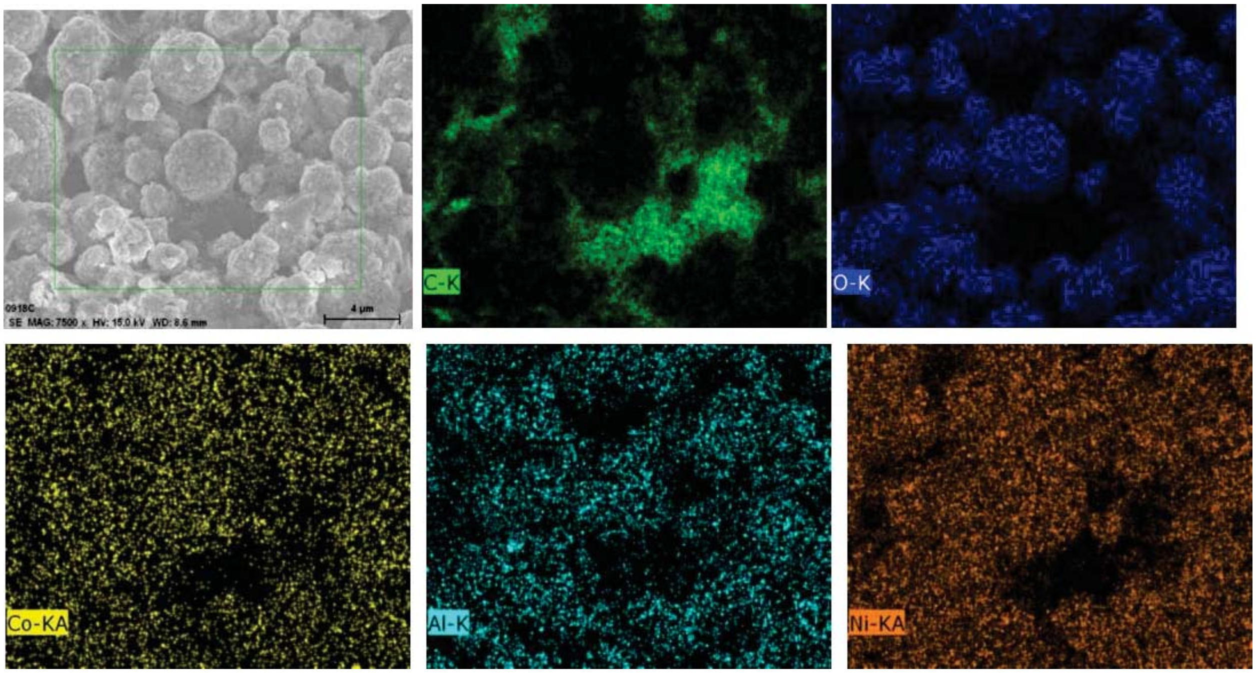

Figure 11: x-ray mapping from NCA-Al2O3 sample

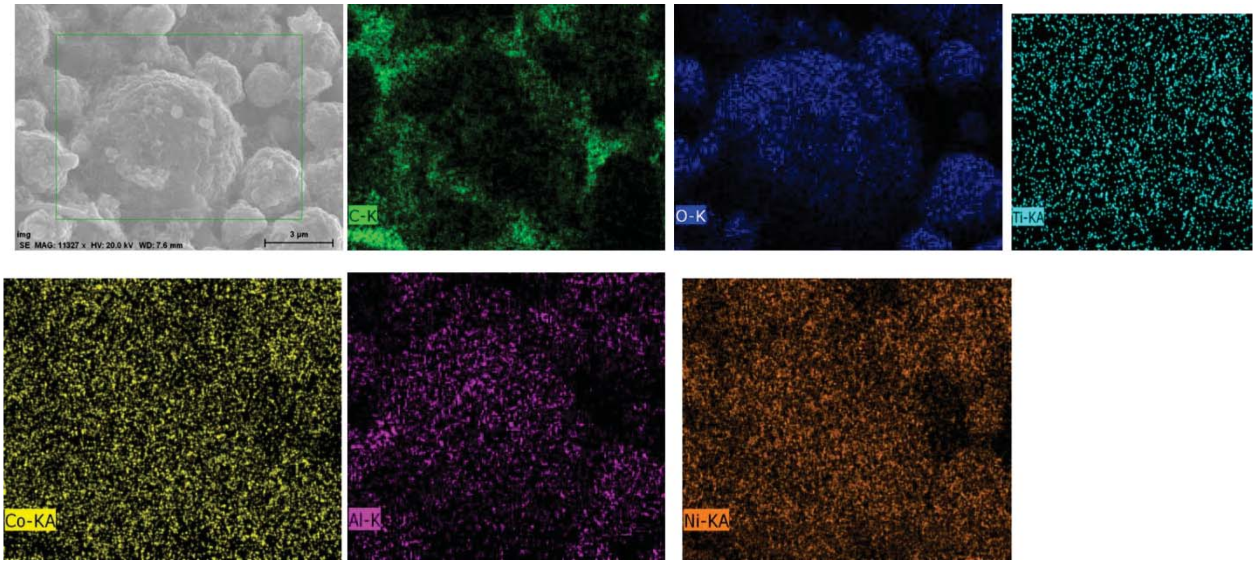

Figure 12: x-ay mapping from NCA-TiO2 sample
